# The association of residential greenness and ambient particulate matter with hearing impairment in Chinese middle-aged and elderly adults: a nationwide cohort study

**DOI:** 10.1007/s40520-025-03002-x

**Published:** 2025-04-07

**Authors:** Jia-min Yan, Min-zhe Zhang, Hong-jie Yu, Qi-qiang He

**Affiliations:** 1 Department of Laboratory Medicine, Wuhan Children’s Hospital (Wuhan Maternal and Child Healthcare Hospital), Tongji Medical College, Huazhong University of Science & Technology, Wuhan, P. R. China; 2 School of Public Health, Wuhan University, Wuhan, China; 3 Hubei Biomass-Resource Chemistry and Environmental Biotechnology Key Laboratory, Wuhan University, Wuhan, China

**Keywords:** Residential greenness, Particulate matter, Hearing impairment, Cohort study

## Abstract

**Objective:**

To examine the association of residential greening and atmospheric particulate matter (PM_2.5_, PM_10_) with the risk of hearing impairment in Chinese middle-aged and elderly adults.

**Methods:**

Data from the China Health and Retirement Longitudinal Study (2011 to 2018 wave) were used. The degree of greening of residential areas were quantified using the Normalized Difference Vegetation Index (NDVI) from the Moderate Resolution Imaging Spectroradiometer (MODIS). PM concentrations were obtained from the CHAP dataset. Hearing impairment was self-reported by the participants. Cox proportional hazards models were used to assess the risk of hearing impairment associated with exposure to residential green spaces and PM. Additionally, the study investigated the potential modifying and mediating role between residential greenery, PM exposure and hearing impairment.

**Results:**

A total of 13,585 participants aged 61.84 years (Standard deviation: 9.17) were included in this study. Over a span of 7 years, 2,527 cases of hearing impairment were reported, with an incidence rate of 18.6%. A higher degree of residential greenness was inversely associated with the risk of hearing impairment, showing a HR of 0.688 (95%CI: 0.659–0.719) for every 0.1 unit increment in NDVI. In contrast, a 10 µg/m^3^ elevation in PM_2.5_ and PM_10_ concentration was linked to a 67.6% (HR: 1.676; 95%CI: 1.625–1.729) and 30.4% (HR: 1.304; 95%CI: 1.284–1.324) increased risk of hearing impairment, respectively. The harmful effects of PM_2.5_ and PM_10_ were attenuated with higher levels of residential greenness. Furthermore, the mediation analysis revealed that PM_2.5_ and PM_10_ played a significant mediating role in the association between residential greenery exposure and hearing impairment, with mediation proportions of 47.91% for PM_2.5_ and 52.83% for PM_10_.

**Conclusions:**

High residential greenness was associated with a reduced risk of hearing impairment, whereas exposure to PM_2.5_ and PM_10_ may increase the risk of hearing impairment. Additionally, residential greenness may modify the relationship between PM exposure and hearing impairment by reducing exposure to PM_2.5_ and PM_10_.

## Introduction

Hearing impairment, ranging from mild hearing deficits to total deafness, is a major health challenge worldwide [1]. The World Health Organization reported that over 1.5 billion individuals, representing around 20% of the total global population, are currently affected by hearing impairment. By 2050, the number of affected individuals will rise above 2.5 billion due to the increasing aging population exposure to risk factors [2]. In China, the number of hearing-impaired people rose from 224.4 million in 1990 to 426.5 million in 2019. By 2034, the estimated number of individuals with hearing impairment will reach 561 million, accounting for over 40% of the Chinese population [3]. The impact of hearing impairment extends beyond the sensory deficit, profoundly influencing communication abilities, educational and occupational opportunities, and social and emotional well-being [4, 5]. Moreover, untreated hearing impairment has been found to contribute to social isolation, depressive symptoms, cognitive impairment, and an elevated risk of dementia [6–8]. Therefore, identifying potential risk factors for hearing impairment is critical in alleviating its burden.

The etiology of hearing impairment is involved a complex interplay of genetic predispositions, environmental exposures, and lifestyle factors [9–11]. In contrast to altering genetic factors, controlling environmental factors including residential greenness and air pollution may be a more feasible strategy to lessen the occurrence of auditory impairment. Residential greenery, often quantified using satellite-based vegetation indices, represents the amount and quality of green spaces in living environments. Extensive research has documented health benefits associated with residential greenness [12, 13]. However, there is limited research examining the link between residential green spaces and hearing impairment risk.

Particulate matter (PM), a pervasive factor of atmospheric pollution, may play a dominant role among air pollutants as it is more strongly associated with all-cause mortality and mortality from non-malignant respiratory diseases than other pollutants [14–16]. Furthermore, PM has been associated with a variety of adverse health outcomes, including cardiovascular diseases, negative neurodevelopmental and cognitive effects [17, 18]. PM exposure may also contribute to hearing impairment by inducing oxidative stress and inflammation within the cochlea, damaging hair cells and auditory neurons [19]. Chronic PM exposure can induce neuroinflammation, disrupting central auditory processing pathways in the brain and exacerbating hearing difficulties [20]. However, research on the link between PM and hearing impairment remains sparse and inconsistent. A cohort study in Taiwan revealed that PM_2.5_ had a notable effect on the occurrence of sudden sensorineural hearing loss (SSHL) [21]. A cross-sectional study in Korea reported that PM_10_ was correlated with an elevated risk of hearing loss, while Lee et al. found no significant correlation between mean or maximum PM_10_ or mean PM_2.5_ and SSHL [22, 23].

High levels of greenness are generally associated with low PM levels, as vegetation can act as a natural filter, capturing and reducing airborne pollutants [24]. Thus, we hypothesized that the effect of PM on hearing impairment would be modified by residential greenness by reducing exposure to PM. Herein, we investigated the impact of chronic exposure to residential greenness and PM air pollution (PM_2.5_ and PM_10_) with the risk of hearing impairment in a cohort of Chinese middle-aged and elderly adults.

## Methods

### Study population

Data for this analysis were sourced from the China Health and Retirement Longitudinal Study (CHARLS), a large-scale national survey dedicated to exploring the health and socioeconomic conditions of middle-aged and elderly individuals aged 45 years and above in China [25]. 17,708 people from 150 counties distributed across 28 provinces of China took part in the baseline survey conducted in 2011–2012. To track long-term trends, follow-up data were systematically obtained every 2–3 years. This study received ethical approval from the Biomedical Ethics Committee of Peking University (IRB00001052–11015). All participants provided written informed consent prior to the research.

The present study included participants who were 45 years or older without hearing impairment in 2011 (baseline) and participated in one or more of the follow-up assessments (2013, 2015 and 2018), while participants with missing exposure measurement data were excluded.

### Assessment of exposure

The extent of residential greenness was quantified using the Normalized Difference Vegetation Index (NDVI), a metric that varies from − 1 to + 1. Higher NDVI values (approaching + 1) reflect a dense presence of vegetation, while values near − 1 correspond to water bodies. The NDVI data were obtained from the MODIS MOD13Q1 product (version 5), which delivers a 16-day composite at a fine spatial resolution of 250 m by 250 m. To assess greenness at the city level, we extracted the annual NDVI data by integrating the MODIS raster images with the administrative division shapefiles for China.

PM_2.5_ and PM_10_ concentrations, measured over an extended period and across distinct geographical regions, were retrieved from the CHAP dataset [26–28]. This dataset integrates data from ground-level monitoring, satellite-based observation, historical atmospheric data reconstruction, and model-generated simulations to estimate annual concentrations of PM_2.5_ and PM_10_, and has been widely used in environmental studies [29, 30]. Further information on the CHAP dataset is available at https://weijing-rs.github.io/product.html. The yearly ambient levels of PM_2.5_ and PM_10_ were extracted using the same method employed for NDVI. Then, the NDVI, PM_2.5_ and PM_10_ exposure levels for the last 1 year before the onset of hearing impairment or loss to follow-up or the conclusion of the study, whichever occurred first, were averaged for each individual [31].

### Assessment of outcome

The respondents answered the question, “How would you rate your hearing?” with five response categories: excellent, very good, good, fair, or poor. Those who selected “poor” were classified as having hearing impairment, consistent with definitions in previous studies [32, 33].

### Covariates

The dataset on covariates encompassed various factors, including gender, age, body mass index (BMI, calculated by dividing weight in kilograms by the square of height in meters), educational background (illiterate, primary school, or secondary school and above), marital status (married versus single or other), and place of residence (rural versus urban). Smoking status (current smoker versus non-smoker), alcohol consumption habits (more than once per month, less than once per month, or non-consumer), and medical history, including hypertension, dyslipidemia, and diabetes, were also recorded. In addition, cooking fuel (solid fuel, clean fuel), average annual temperature, and relative humidity were included, as these factors have been described in previous studies as influencing hearing impairment [33, 34]. The temperature and relative humidity data were retrieved from the National Earth System Science Data Center, affiliated with the National Science & Technology Infrastructure of China (http://www.geodata.cn). For consistency, we applied the same method used to calculate NDVI when determining the long-term annual temperature and humidity averages.

### Statistical analysis

The baseline characteristics of participants, stratified by the presence or absence of hearing impairment, were summarized using appropriate statistical metrics. Continuous variables were expressed as mean values with standard deviations (mean ± SD), while categorical variables were presented by their frequencies and the corresponding percentages. To evaluate differences between groups, chi-square tests were applied for categorical variables to assess the distribution across groups, and independent sample t-tests were used for continuous variables to compare group means.

Time-varying Cox proportional hazards regression models, with follow-up as the timescale, were utilized to examine the association between residential greenness, PM, and the risk of hearing impairment, with results presented as hazard ratios (HR) and their corresponding 95% confidence intervals (CI). Model 1 was unadjusted, while model 2 adjusted for a wide range of potential confounders, including demographic factors (age, sex, education level, marital status), lifestyle behaviors (smoking and drinking status), health conditions (hypertension, dyslipidemia, diabetes), place of residence, cooking fuel type, BMI, and environmental factors (average annual temperature and relative humidity). For analyses treating exposures as continuous, the HR (95%CI) were calculated per 0.1 unit increase in NDVI and per 10 µg/m^3^ increase in PM_2.5_ or PM_10_ concentrations, allowing for the assessment of exposure effects at fine increments. For categorical analyses, the exposures were divided into tertiles, and HR (95% CI) were reported for each tertile, using the lowest tertile (Tertile 1) as the reference category. To explore potential non-linear relationships between the exposures (residential greenness, PM_2.5_, and PM_10_) and the risk of hearing impairment, restricted cubic spline models with three degrees of freedom were applied.

Subgroup analyses were conducted to explore potential modifications by age (< 65 years, ≥ 65 years), sex (male, female), education level (illiterate, primary school, middle school or above), residence (rural, urban), smoking status (no, yes), and hypertension (no, yes), respectively. *P* values for the interaction between exposure and covariates were calculated using likelihood ratio tests.

We also assessed the potential modifying role of residential greenness on the association between PM_2.5_ and PM_10_ and incident hearing impairment was assessed. Specifically, the effect of continuous PM_2.5_ and PM_10_ exposure was investigated in stratified residential greenness level.

Mediation analysis was conducted to examine the mediating role of PM_2.5_ and PM_10_ in the association between residential greenness and hearing impairment, according to the method proposed by Huang et al. [35]. A linear regression model was used to estimate the relationship between residential greenness and PM concentrations, while a Cox proportional hazard model was applied to evaluate the direct and indirect effects of residential greenness on hearing impairment. HR with 95%CI were calculated for the natural direct effect, natural indirect effect, and total effect. The mediation proportion was determined as the ratio of the indirect effect to the total effect.

All statistical analyses were performed using R version 4.3.2. Statistical significance was defined as a two-sided *P* value less than 0.05.

## Results

Among the 13,585 participants included in this study, 2,527 were identified with hearing impairment during the 7-year follow-up period. Table 1 provides a detailed overview of the baseline characteristics of the study population. The average age of the participants was 61.84 ± 9.17 years and 7,078 (52.1%) were female. Compared to participants without hearing impairment, participants with hearing impairment tended to be older, female, had lower educational attainment, were single, resided in rural areas, cooked with solid fuel, and had a lower BMI and higher prevalence of hypertension.

**Table 1:** Characteristics of study participants stratified by outcome

Characteristics	Total	Non-cases	Cases	*P* value
	*N* = 13,585	*N* = 11,058	*N* = 2527	
**Age**, ** years(M ± SD)**	61.84 ± 9.17	61.11 ± 8.84	65.02 ± 9.90	< 0.001
**Sex**, ***n*(%)**				< 0.001
Male	6497 (47.9)	5370 (48.6)	1127 (44.7)	
Female	7078 (52.1)	5681 (51.4)	1397 (55.3)	
**Education level**, ***n*(%)**				< 0.001
Illiterate	3348 (24.7)	2437 (22.1)	911 (36.1)	
Primary school	5404 (39.8)	4368 (39.5)	1036 (41.0)	
Middle school or above	4820 (35.5)	4242 (38.4)	578 (22.9)	
**Marital status**, ***n*(%)**				< 0.001
Married	12,155 (89.5)	9994 (90.4)	2161 (85.5)	
Single or other	1430 (10.5)	1064 (9.6)	366 (14.5)	
**Residence**, ***n*(%)**				< 0.001
Rural	8202 (60.4)	6447 (58.3)	1755 (69.4)	
Urban	5383 (39.6)	4611 (41.7)	772 (30.6)	
**Smoking status**, ***n*(%)**				0.310
Yes	5314 (39.1)	4349 (39.3)	965 (38.2)	
No	8267 (60.9)	6707 (60.7)	1560 (61.8)	
**Drinking status**, ***n*(%)**				< 0.001
> 1 times per month	3494 (25.7)	2897 (26.2)	597 (23.7)	
< 1 times per month	1115 (8.2)	956 (8.7)	159 (6.3)	
Non-consumers	8965 (66.0)	7197 (65.1)	1768 (70.0)	
**Hypertension**, ***n*(%)**				< 0.001
Yes	3108 (23.0)	2441 (22.2)	667 (26.6)	
No	10,404 (77.0)	8559 (77.8)	1845 (73.4)	
**Dyslipidemia**, ***n*(%)**				0.508
Yes	1202 (9.0)	998 (9.1)	214 (8.7)	
No	12,116 (91.0)	9859 (90.9)	2257 (91.3)	
**Diabetes**, ***n*(%)**				0.369
Yes	713 (5.3)	571 (5.2)	142 (5.7)	
No	12,747 (94.7)	10,389 (94.8)	2358 (94.3)	
**Cooking fuel**, ***n*(%)**				< 0.001
Solid fuel	7221 (53.4)	5638 (51.3)	1583 (62.9)	
Clean fuel	6292 (46.6)	5360 (48.7)	932 (37.1)	
**BMI (M ± SD)**	23.59 ± 3.77	23.68 ± 3.76	23.19 ± 3.82	< 0.001

Abbreviation: M ± SD, Mean ± Standard deviation; BMI, body mass index. *P* values were obtained using the t-test for continuous variables and the Chi-square test for categorical variables

The associations between NDVI, PM_2.5_, PM_10_ levels, and hearing impairment risk are summarized in Table 2. In the fully adjusted model, an increase of 0.1 unit in NDVI may be linked to a 31.2% decrease in hearing impairment risk (HR: 0.688; 95%CI: 0.659–0.719). Individuals in the group with the highest NDVI tertile had a 53.3% lower risk of hearing impairment than those in the group with the lowest tertile (HR: 0.467; 95%CI: 0.418–0.522). Conversely, higher concentrations of PM_2.5_ and PM_10_ were linked to a higher risk of hearing impairment. Specifically, an increase in PM_2.5_ concentration of 10 µg/m^3^ was corresponded to a 67.6% higher risk (HR: 1.676; 95%CI: 1.625–1.729), while an increase in PM_10_ concentration of 10 µg/m^3^ may result in a 30.4% increased risk (HR: 1.304; 95%CI: 1.284–1.324). Furthermore, participants in the group with the highest tertile of PM_2.5_ exposure had a 382.6% increased risk of hearing impairment than that of those in the group with the lowest tertile (HR: 4.826; 95%CI: 4.235–5.478). Similarly, those in the group with the highest tertile of PM_10_ exposure had 391.8% increased risk compared to those in the group with the lowest tertile (HR: 4.918; 95%CI: 4.342–5.569).

**Table 2:** Association of residential greenness and particulate matter with risk of hearing impairment

Exposure	Number of cases/Total	Model 1 (HR 95%CI)	Model 2 (HR 95%CI)
NDVI			
Continuous (per 0.1 unit increase)	2527/13,585	0.761 (0.732,0.790)	0.688 (0.659,0.719)
Tertile 1	1013/4529	Ref	Ref
Tertile 2	842/4528	0.774 (0.706,0.848)	0.626 (0.559,0.702)
Tertile 3	672/4528	0.573 (0.520,0.632)	0.467 (0.418,0.522)
*P* for trend		< 0.001	< 0.001
PM_2.5_			
Continuous (per 10 ug/m^3^ increase)	2527/13,585	1.302 (1.271,1.333)	1.676 (1.625,1.729)
Tertile 1	627/4529	Ref	Ref
Tertile 2	770/4528	1.290 (1.161,1.433)	1.712 (1.513,1.937)
Tertile 3	1130/4528	2.133 (1.934,2.352)	4.826 (4.235,5.478)
*P* for trend		< 0.001	< 0.001
PM_10_			
Continuous (per 10 ug/m^3^ increase)	2527/13,585	1.102 (1.090,1.114)	1.304 (1.284,1.324)
Tertile 1	716/4529	Ref	Ref
Tertile 2	677/4528	0.954 (0.859,1.060)	1.372 (1.212,1.554)
Tertile 3	1134/4528	1.824 (1.661,2.003)	4.918 (4.342,5.569)
*P* for trend		< 0.001	< 0.001

Abbreviation: HR, hazard ratio; CI, confidence interval; PM_2.5_, particulate matter ≤ 2.5 μm; PM_10_, particulate matter ≤ 10 μm; NDVI, Normalized Difference Vegetation Index

Model 1: Unadjusted model

Model 2: Adjusted for age (< 65 years, ≥ 65 years), sex (male, female), education level (illiterate, primary school, middle school or above), marital status (married, single or other), residence (rural, urban), smoking status (yes, no), drinking status (drink more than once a month, drink but less than once a month, none of these), hypertension (yes, no), dyslipidemia (yes, no), diabetes (yes, no), BMI (kg/m^2^), and cooking fuel (solid fuel, clean fuel), temperature and humidity

**Fig. 1:**
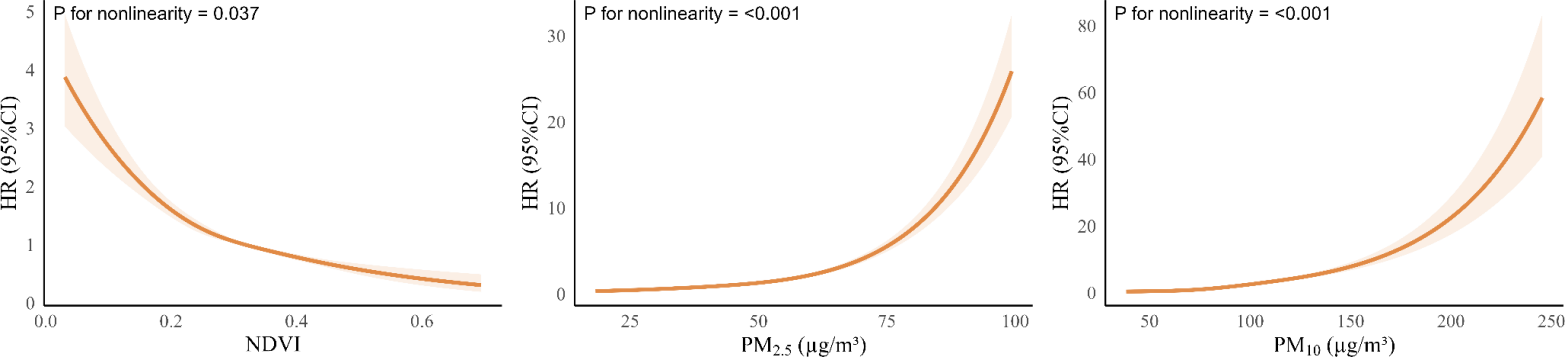
Concentration-response curves for the association of residential greenness and particulate matter with risk of hearing impairment using restrict cubic splines

Figure 1 illustrates the curves depicting the dose-response relationships between NDVI, PM and hearing impairment. We observed a negative and non-linear exposure-response curve between NDVI and hearing impairment. In contrast, the curves for PM_2.5_ and PM_10_ were positive and non-linear, with steeper slopes observed at concentrations exceeding 75 µg/m^3^ and 200 µg/m^3^, respectively.

**Fig. 2:**
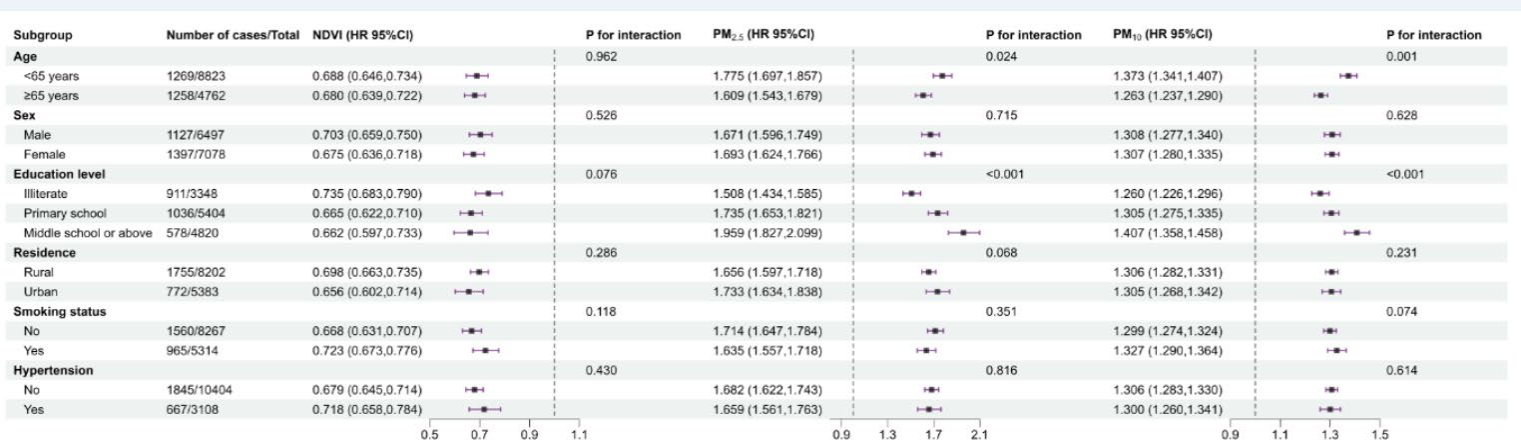
Subgroup-specific HR estimates (95%CI) of hearing impairment associated with annual average exposure to environmental factors

The results of the subgroup analyses examining the associations between residential greenness, PM_2.5_ and PM_10_ with hearing impairment are shown in Fig. 2. Stronger associations between exposure to PM_2.5_ and PM_10_ and the risk of hearing impairment were found among the younger and high educated participants (*P* for interaction < 0.05). No significant difference was found in the association between NDVI and the risk of hearing impairment in the different subgroups.

**Fig. 3:**
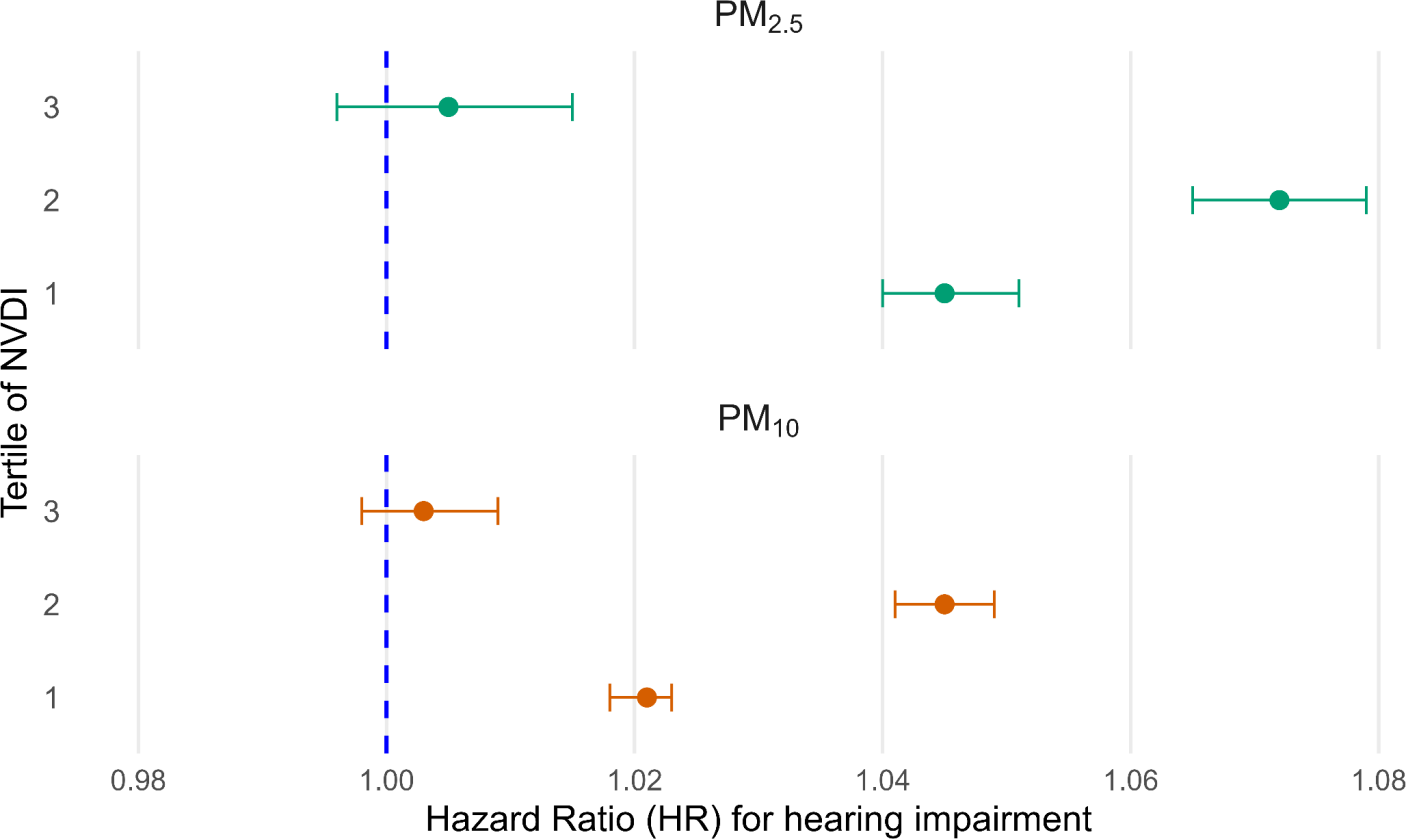
Associations of particulate matter with risk of hearing impairment stratified by different levels of residential greenness (in tertiles)

The modification effect of residential greenness on the relationship between PM_2.5_, PM_10_, and the risk of hearing impairment is shown in Fig. 3. Significant associations between PM_2.5_, PM_10_ and risk of hearing impairment were observed in the groups with low NDVI (tertile 1) and medium NDVI (tertile 2), but not in the high NDVI (tertile 3) group. The HR (95%CI) for an increase of 1 µg/m^3^ in PM_2.5_ and PM_10_ in the high NDVI group was 1.005 (0.996–1.015) and 1.003 (0.998–1.009), respectively.

Table 3 illustrates the mediating role of PM_2.5_ and PM_10_ in the relationship between residential greenness and hearing impairment. Specifically, PM_2.5_ and PM_10_ accounted for 47.91% and 52.83% of the mediation effect, respectively.

**Table 3:** Mediation effects of particulate matter on the association between residential greenness and risk of hearing impairment

Particulate matter	Direct effect (HR, 95%CI)	Indirect effect (HR, 95%CI)	Total effect (HR, 95%CI)	Proportion mediated
PM_2.5_	0.916 (0.898,0.933)	0.922 (0.913,0.931)	0.844 (0.826,0.862)	47.91%
PM_10_	0.927 (0.909,0.945)	0.919 (0.908,0.929)	0.852 (0.833,0.871)	52.83%

Abbreviation: HR, hazard ratio; CI, confidence interval; PM_2.5_, particulate matter ≤ 2.5 μm; PM_10_, particulate matter ≤ 10 μm

Mediation model was adjusted for age (< 65 years, ≥ 65 years), sex (male, female), education level (illiterate, primary school, middle school or above), marital status (married, single or other), residence (rural, urban), smoking status (yes, no), drinking status (drink more than once a month, drink but less than once a month, none of these), hypertension (yes, no), dyslipidemia (yes, no), diabetes (yes, no), BMI (kg/m^2^), and cooking fuel (solid fuel, clean fuel), temperature and humidity

## Discussion

To the best of our knowledge, this study represents the first comprehensive investigation of the synergistic effects of PM and residential greenness on hearing impairment in a large, population-based cohort. Our findings suggest that a high level of residential greenness may play a protective role against hearing impairment. In contrast, exposure to PM_2.5_ and PM_10_ was positively correlated with an increased risk of hearing impairment. Notably, the protective benefits of residential greenness appeared to be consistent across different subgroups, with no significant variations observed across demographic categories. In contrast, the detrimental effects of PM_2.5_ and PM_10_ levels on the risk of hearing impairment were significantly pronounced among younger individuals and those with higher educational attainment. Furthermore, our analysis revealed a modifying effect of residential greenness on the relationship between PM_2.5_ and PM_10_ exposure and the risk of hearing impairment. Mediation analysis further suggested that the beneficial impact of residential greenness could be partially attributed to its role in reducing exposure to harmful PM, providing an indirect pathway through which greenness mitigates hearing impairment risk.

To date, only one study has examined the association between residential greenness and the risk of hearing impairment. A cross-sectional study from the UK Biobank, including over 100,000 participants found that each interquartile increase in the residential greenness was linked to a 19% reduction in the odd of hearing impairment (OR: 0.81; 95%CI: 0.79–0.83) [36]. Our study using a longitudinal design suggested that a 0.1 unit increase in NDVI was associated with a 31.2% lower risk of incident hearing impairment, thus providing further evidence related residential greenness with hearing health.

There are limited studies on the relationship between PM exposure and the risk of hearing impairment and the results yield inconsistent findings. A cross-sectional study involving 158,811 participants aged from 37 to 73 years in the UK Biobank found that PM_10_, but not PM_2.5_, was significantly associated with hearing impairment (OR: 1.15; 95%CI: 1.02–1.30) [37], while a nested case-control study from Korea reported no significant association between PM_10_ exposure and the risk of SSHL [38], and another observational study in Taipei identified a positive association between PM_2.5_ exposure and an elevated risk of SSHL (RR: 1.195; 95%CI: 1.047–1.363) [39]. The discrepancies observed in various studies may be a result of differences in the characteristics of the study population, research design, sample sizes, and different approaches used to assess PM exposure. Thus, further study with large samples and long follow-up period are warranted to confirm our results.

There was a strong association of PM with hearing impairment in the present study in comparison with previous studies. For example, a study conducted in Korea found that individuals exposed to PM_10_ had a 10% and 11% higher risk of hearing impairment at speech-frequency and high-frequency, respectively [22]. In contrast, our study found that exposure to PM_10_ was associated with a 30.4% increased risk of hearing impairment. This could be due to the higher PM concentrations in China (PM_10_: 87.8 µg/m^3^ in our study) compared to Korean (48.22 µg/m^3^) [40]. Furthermore, our study focuses specifically on middle-aged and older adults, who are more likely to suffer from comorbidities, and have an increased vulnerability to the negative effects of air pollution on hearing health.

The associations of residential greenness and PM with hearing impairment can be explained by several mechanisms. Prolonged exposure to elevated concentrations of PM_2.5_ and PM_10_ can cause systemic inflammation and vascular damage in the microvasculature of the auditory system [41], which may impair blood flow to the cochlea and lead to hearing impairment. The beneficial effects of residential greenness may be due to its influence on promoting physical activity, reducing stress, and fostering social interactions, all of which could contribute to better overall health and potentially lower the risk of hearing impairment [42, 43]. In addition, findings from our mediation analysis indicate that reductions in PM concentrations may serve as a partial mechanism linking residential greenness to a lower risk of hearing impairment. Residential greenness is often associated with lower PM levels due to the ability of vegetation to filter and trap PM_2.5_ and PM_10_ [24]. We also found that the mediation effect of PM_10_ was larger than PM_2.5_ in the link between NDVI and hearing impairment. This may because that PM_10_ can settle more easily on leaves and other vegetation surfaces than PM_2.5_ due to its larger size and mass.

The stratified analyses indicate that a stronger association of PM_2.5_ and PM_10_ exposure with the risk of hearing impairment was observed in younger and more educated individuals. This could be explained by their higher likelihood of residing in urban settings and engaging in outdoor activities, such as commuting or working in high-traffic environments, with high exposure to air pollution.

This study has several strengths. First, it leverages data from a large-scale, nationwide sample, which extends the applicability of our findings to the middle-aged and elderly population across China. Second, we employed robust statistical methods, including Cox proportional hazards models and mediation analyses, to assess both direct and indirect pathways, providing a holistic view of the relationships between residential greenness, PM pollution, and hearing impairment. Lastly, the use of high-resolution satellite data for assessing residential greenness and well-validated datasets for PM concentrations strengthened the accuracy and reliability of our exposure measurements.

However, several limitations should be considered in this study. First, hearing impairment was assessed via self-report, which may introduce recall bias or misclassification. Objective audiometric testing would provide more precise measurement of hearing impairment. Second, although we controlled for a broad range of potential confounders, residual confounding cannot be fully excluded, particularly from unmeasured variables such as occupational noise exposure or genetic predispositions. Third, the relevance of our findings may not extend to younger populations or other countries with different environmental and sociodemographic contexts.

## Conclusion

In conclusion, our study provides evidence that PM was significantly associated with an increased risk of hearing impairment, while residential greenness may decrease the risk of hearing impairment, potentially through the reduction of exposure to PM pollution. Future research should include objective measures of hearing impairment and explore these associations in different populations and settings to validate our findings.

## Data Availability

The data of this study can be obtained on the official website of http://www.charls.pku.edu.cn/.
